# Accelerated Degradation Test and Predictive Failure Analysis of B10 Copper-Nickel Alloy under Marine Environmental Conditions

**DOI:** 10.3390/ma8095290

**Published:** 2015-09-10

**Authors:** Bo Sun, Tianyuan Ye, Qiang Feng, Jinghua Yao, Mumeng Wei

**Affiliations:** 1 School of Reliability and Systems Engineering, Beihang University, Beijing 100191, China; E-Mails: sunbo@buaa.edu.cn (B.S.); yetianyuan@buaa.edu.cn (T.Y.); 2 State Key Laboratory for Marine Corrosion and Protection, Luoyang 471000, China; E-Mails: yaolele725@163.com (J.Y.); syamm2011@163.com (M.W.)

**Keywords:** copper alloy, failure analysis, corrosion, marine environmental, accelerated degradation test

## Abstract

This paper studies the corrosion behavior of B10 copper-nickel alloy in marine environment. Accelerated degradation test under marine environmental conditions was designed and performed based on the accelerated testing principle and the corrosion degradation mechanism. With the prolongation of marine corrosion time, the thickness of Cu_2_O film increased gradually. Its corrosion product was Cu_2_(OH)_3_Cl, which increased in quantity over time. Cl^−^ was the major factor responsible for the marine corrosion of copper and copper alloy. Through the nonlinear fitting of corrosion rate and corrosion quantity (corrosion weight loss), degradation data of different corrosion cycles, the quantitative effects of two major factors, *i.e.*, dissolved oxygen (DO) and corrosion medium temperature, on corrosion behavior of copper alloy were analyzed. The corrosion failure prediction models under different ambient conditions were built. One-day corrosion weight loss under oxygenated stirring conditions was equivalent to 1.31-day weight loss under stationary conditions, and the corrosion rate under oxygenated conditions was 1.31 times higher than that under stationary conditions. In addition, corrosion medium temperature had a significant effect on the corrosion of B10 copper sheet.

## 1. Introduction

B10 copper-nickel alloy (which contains 90% copper and 10% nickel) is widely used in the condenser tubes of various ships, heat exchangers of coastal power plants and piping systems of seawater desalination equipment owing to its outstanding resistance to corrosion, particularly in flowing seawater. Copper-nickel will gradually replace copper to become the mainstream piping material. Many studies have been carried out on copper-nickel alloy, such as corrosion behavior under various environmental conditions, extension of lifecycle, and improvement of corrosion resistance [1,2,3,4,5,6]. Efird and Anderson collected fourteen years data for 90–10 and 70–30 cupronickel alloys exposed in seawater at the LaQue laboratory, including corrosion rates for both alloys in quiet and flowing water as well as in the tidal zone [7]. Parvizi *et al.* studied the behavior and protection mechanism of the alloy under differing flow velocities, temperatures, pH values, compositions of seawater, and many other factors [8]. Kear *et al.* believed that copper alloys can form a layer of oxide film during the early stage of immersion in marine water, and their corrosion resistance depends on such surface film layer under relatively stable solution environment, dissolved oxygen and temperature conditions [9]. Melchers pointed out that there are apparently conflicting observations about the influence of seawater temperature on the immersion corrosion of copper-nickel alloys, and he proposed the relationship between corrosion and temperature in the range of 10 °C to 40 °C [10]. More recently, he described the maximum corrosion depth of copper alloy by marine corrosion using bi-modal and power functions [11]. Chen studied the corrosion behavior of copper alloy in 3.5% NaCl solution [12]. Arjmand *et al.* studied the effects of pH and chloride concentration on corrosion behavior of copper alloy, and observed significant macroscopic and microscopic differences in the copper alloy before and after corrosion in NaCl solution [13]. 

In the above-mentioned studies, most scholars studied the marine environmental influencing factors using artificial seawater (3.5% NaCl solution) as the corrosive medium [14], which ignores the complex metal ion components in marine water, and somewhat differs from natural seawater corrosion. Especially, experiments usually require a long and costly testing process, and it has been found to result in more qualitative results. At present, research on corrosion law and property degradation of copper alloys by natural seawater under different environmental factors, particularly with quantitative conclusions, are relatively scant. Additionally, in the field of engineering failure analysis, the analysis is usually carried out after the failure occurs. To effectively prevent the risk of accidents, predictive failure analysis is necessary. The prerequisites for conducting predictive failure analysis are the results of quantitative analysis and the building of quantitative mathematical models.

This paper deals with a laboratory test in natural marine environment based on the accelerated testing principle and the corrosion degradation mechanism in Section 2. Section 3 studies the changes in surface film and micro morphology of B10 copper-nickel alloy at different corrosion cycles, and analyzes its corrosion products and corrosion mechanism. Section 4 further deals with an experiment to study the effects of different medium temperatures and dissolved oxygen on copper alloy corrosion behavior from the perspective of corrosion rate changes. Section 5 builds the corrosion prediction models under different environmental conditions and conducts relevant discussion.

## 2. Accelerated Degradation Test

The test sample used in this study was B10 copper-nickel alloy sheet, and its chemical composition is shown in Table 1. Samples used in the full-immersion simulated accelerated test had a size of 100 mm × 50 mm × 3 mm, and silvery white surface. After perforation and marking, the samples were measured for length, width and thickness, washed with anhydrous ethanol, dried for 24 h in a dryer, and weighed. Then, the samples were immersed in natural seawater solution for full-immersion test, where 0.05 mol/L H_2_O_2_ was added as the acceleration means, and the corrosion medium temperature was controlled at 30 °C using a constant temperature water bath. Test cycles included nine cycles: one day, three days, one week, two weeks, three weeks, four weeks, six weeks, eight weeks and 16 weeks. Four parallel samples were used at each cycle, and the test solution was replaced every three days.

**Table 1:** Chemical composition (wt %) of B10 copper-nickel alloy.

Si	Cu	P	Fe	S	Zn	Ni	Mn
<0.15	Allowance	<0.02	1.20	0.0013	<0.3	9.76	0.74

Samples taken out at various cycles were subjected to surface film observation, micro morphology observation, corrosion product analysis and corrosion quantity/rate determination, respectively. In the surface film observation, the surface microscopic images of samples were observed by scanning electron microscopy (SEM), and the material surface elements of samples were identified by energy-dispersive X-ray spectroscopy (EDS). In the micro morphology observation, surface film was observed and analyzed after removing corrosion products from the sample surface. In the corrosion product analysis, the elemental composition, electronic structure, chemical bonds and elements of material surface substances were analyzed in depth using X-ray photoelectron spectroscopy (XPS). Besides, changes in the structure of surface adsorbed molecules were observed by Raman spectroscopy. Among the four sets of parallel samples, one set was used for surface film and micro morphology observation, while the remaining three sets were used for the measurement of corrosion quantity and corrosion rate.

## 3. Observation Results and Analysis

### 3.1. Surface Film Observation

B10 copper-nickel alloy would form a layer of oxide film when immersed in seawater, whose forming quality, density and breakage had significant effects on the marine corrosion resistance of B10 copper-nickel alloy.

**Figure 1:**
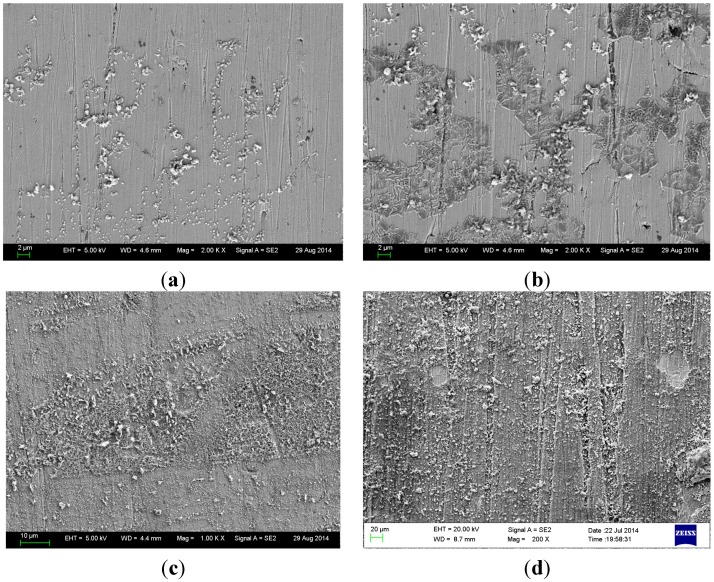
SEM photographs of B10 alloy surface film at different corrosion times: (**a**) initial surface; (**b**) one day; (**c**) three days; and (**d**) one week.

Macroscopic morphology of B10 was observed during the test. Clean B10 copper sheet surface was silvery white, and after corrosion, it would continuously form an oxide film. Figure 1 shows SEM photographs of B10 copper-nickel alloy surface film at different corrosion times. Among them, Figure 1a is the initial morphology of B10 sample, which clearly shows the polished streaks of copper sheet. Very small particulate matters were attached on the sample surface, which were distributed at large gaps and yet to be connected in pieces. After corroding for one day, as shown in Figure 1b, although there were still some particles on the sample surface, they were significantly enlarged compared to the initial sample. Moreover, small pieces of flat oxide film were gradually formed in local areas. Surface status of sample was still visible, and the substrate surface was not completely covered by the corrosion product film. The film was in the growth phase. Figure 1c shows the surface morphology after corroding for three days. Small pieces of oxide film had begun to be attached on the sample surface in blocks, and the oxide film was loose and porous at that time. One week later, as shown in Figure 1d, the oxide film was already intact and rather dense, with a thick layer of corrosion product attached to it. This process showed that B10 copper sheet could generate a thin surface film just in the initial stage of marine corrosion, yet the oxide film formed lacked integrity and had many defects. With the progression of corrosion reaction, corrosion product film would gradually become intact and dense.

EDS analysis was performed on surfaces immersed for different times, and the results are shown in Table 2. As can be seen, surface films formed by corrosion of B10 copper sheet for different times contained a relatively high content of oxygen whose composition was mainly oxide apart from containing copper, nickel, *etc.* that copper alloy substrates originally contain. Nickel content declined slightly, while the oxygen content gradually increased as the corrosion reaction proceeded, indicating the continuation of oxidation reaction on sample surface with the extension of corrosion time.

**Table 2:** EDS analysis of surface film (unit: %).

Element	Original	1 Day	3 Day	7 Day
Cu	79.93	72.56	67.19	59.71
Ni	10.4	9.2	8.63	2.47
O	9.55	18.24	22.95	27.8
Cl	0.12	0.25	1.23	10.02

### 3.2. Micromorphology Observation

After full-immersion in indoor simulated seawater and accelerated corrosion of B10 copper sheets for different cycles, the samples were immersed in 10-fold diluted sulfuric acid solution with a relative density of 1.84 for 3 min. Then, the corrosion products were removed with a stiff brush, and the sample surfaces were subjected to micro morphology observation by SEM.

**Figure 2:**
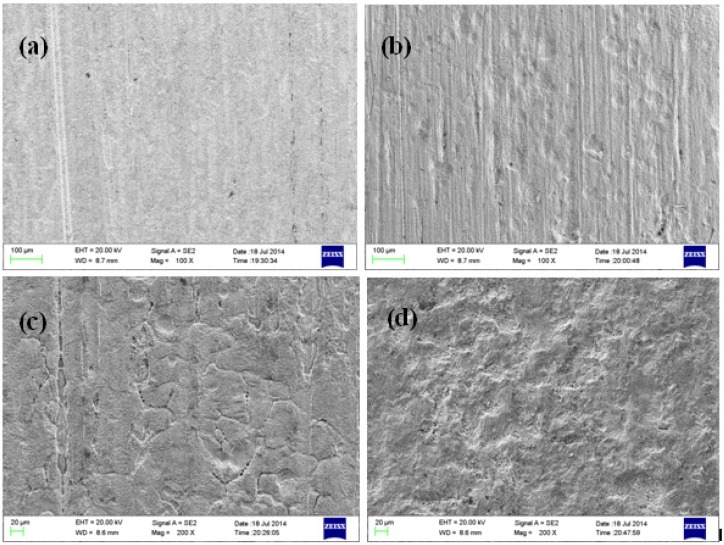
Microscopic corrosion morphology of B10 alloy at different corrosion times: (**a**) one week; (**b**) two weeks; (**c**) four weeks; and (**d**) eight weeks.

Figure 2 shows the microscopic corrosion morphology of B10 copper sheet corroded in seawater for different times after removing the corrosion products. As can be seen from the figure, with the extension of corrosion time, small and shallow corrosion pits appeared gradually on the surface of B10 copper sheet, which was originally rather smooth. On the eighth week, patchy corrosion was clearly visible, with possible corrosion exfoliation in local areas. EDS analysis on surface composition revealed that the proportion of Cu to Ni contents in B10 samples increased from 90/10 to 81.34/4.86 after eight weeks of corrosion, suggesting the occurrence of de-nickel corrosion.

### 3.3. Analysis of Corrosion Products

**Figure 3:**
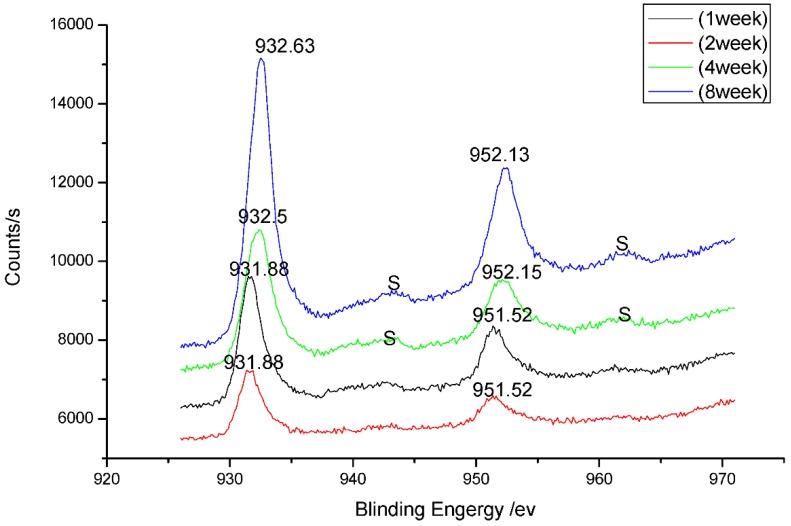
Cu2p XPS narrow spectrogram of B10 alloy corrosion products at one, two, four and eight weeks.

**Figure 4:**
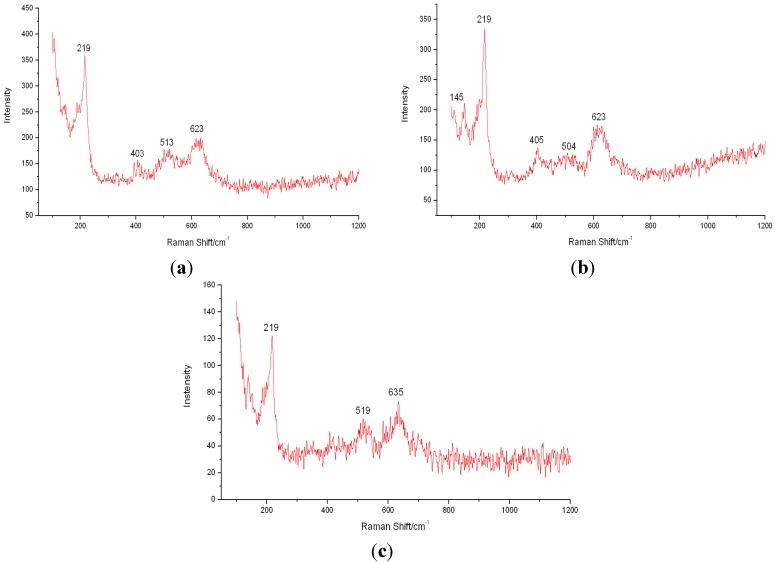
Raman spectra of B10 alloy corrosion products at (**a**) one week; (**b**) two weeks; and (**c**) four weeks.

The corrosion products of B10 copper-nickel alloy at corrosion different cycles were analyzed by XPS and Raman spectroscopy. Figure 3 shows the Cu2p XPS narrow spectrogram of corrosion products after one, two, four and eight weeks of B10 copper alloy corrosion, while Figure 4 is the Raman spectra after one, two, and four weeks of B10 corrosion.

As can be seen from Figure 3, due to the spin-orbit coupling, Cu 2p energy level split, and two peaks, Cu 2p3/2 and Cu 2p3/2, emerged in the spectrogram, with positions of 931.88–932.63.0 eV and 951.52–952.15 eV, respectively. With the extension of marine corrosion time, a weak vibration excitation peak S appeared at the high binding energy end of primary photon beams, as shown in Figure 3, which became intense over time. Since Cu and Cu_2_O series compounds did not have the vibration excitation peak of Cu2p3/2 line, and as can be seen from the Raman spectroscopic analysis on one-week corrosion products, peak positions were at 219 cm^−1^ and 513 cm^−1^ in the spectrogram. Therefore, Cu_2_O should be contained in the surface layer oxide of the sample after corrosion. As can be from the intensity of peak in the figure, Cu_2_O film was gradually thickened with the extension of marine corrosion time. Presence of divalent copper could be inferred from the corrosion products where vibration excitation peaks occurred, and generation of Cu_2_(OH)_3_Cl could be inferred based on the occurrence of peak positions 219 cm^−1^, 623 cm^−1^ (two weeks), 219 cm^−1^ and 635 cm^−1^ (four weeks) of corrosion products at different times in the Raman spectra. It could be seen from the peak intensity that this product increased continuously over time.

### 3.4. Analysis of Corrosion Mechanism

From the perspective of chemical reaction mechanisms, thermodynamic view indicated that after Cu atom on the B10 copper-nickel alloy surface lost an electron, it left the metal surface into the medium in the form of Cu^+^, $\text{Cu}\to {\text{Cu}}^{+}+e$.

Cu^+^ was diffused on the surface of B10 samples, contacted with corrosive factors OH^−^ and Cl^−^, and produced complex reaction. The reaction products may include Cu_2_O, CuCl and CuCl_2_^−^, which were $2{\text{Cu}}^{+}+2{\text{OH}}^{-}\to {\text{Cu}}_{2}\text{O}+{\text{H}}_{2}\text{O}$, ${\text{Cu}}^{+}+{\text{Cl}}^{-}\to \text{CuCl}$. Cuprous chloride continued to react with chloride ions to generate more sophisticated product $\text{CuCl}+{\text{Cl}}^{-}\to {{\text{CuCl}}_{2}}^{-}$.

At the same concentration, Cl^−^ could more easily react with Cu^+^ than OH^−^; moreover, Cl^−^ concentration was far higher than OH^−^ concentration in natural seawater, so Cl^−^ was the main corrosive factor responsible for marine corrosion of copper and copper alloy. As CuCl_2_^−^ was easily soluble, yellow-green water film could easily be formed on the sample surface. During the early stage of corrosion reaction, Cu would be oxidized and dissolved in the monovalent form to produce de-copper corrosion of alloy. With the dissolution of copper, Cl^−^ concentration at the interface of alloy and seawater markedly decreased, making continuation of $\text{CuCl}+{\text{Cl}}^{-}\to {{\text{CuCl}}_{2}}^{-}$ impossible. CuCl would also, in turn, be deposited on the alloy surface, and then hydrolyzed to form thermodynamically more stable monovalent copper oxide Cu_2_O, $2\text{CuCl}+{\text{H}}_{2}\text{O}\to {\text{Cu}}_{2}\text{O}+2\text{HCl}$. The Cu_2_O generated attached on the sample surface to form a reddish brown oxide film.

Since Cu_2_O had P-type semiconductor defect loose structure, when the reaction proceeded to a certain stage, the defect sites of oxide film reacted with the dissolved oxygen and chlorine ions in seawater to produce loose and porous blue-green basic copper chloride, $2{\text{Cu}}_{2}\text{O}+{\text{O}}_{2}+2{\text{Cl}}^{-}+4{\text{H}}_{2}\text{O}\to 2{\text{Cu}}_{2}{\text{(OH)}}_{3}\text{Cl}+2{\text{OH}}^{-}$, and pitting or patchy corrosion began to occur. This agreed with the above XPS and Raman analysis results.

## 4. Experiments and Consideration on Environmental Effects 

### 4.1. Effect of Dissolved Oxygen

In order to study the effect of dissolved oxygen on the corrosion behavior of B10 alloy, a control group where electromagnetic air pump was used for oxygenation and stirring was set up on the basis of the above tests, while the other test conditions remained unchanged.

**Figure 5:**
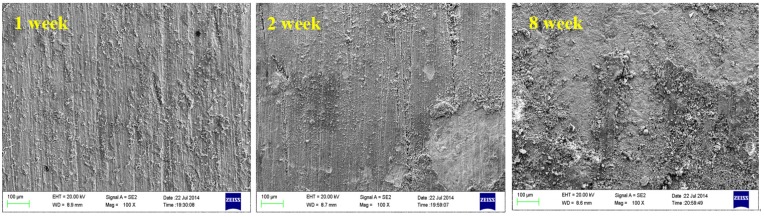
Corrosion morphology of B10 alloy under stationary conditions.

**Figure 6:**
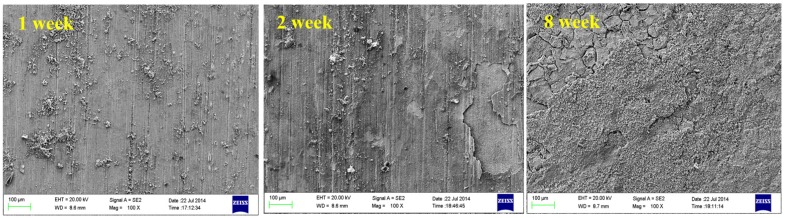
Corrosion morphology of B10 alloy under oxygenated stirring conditions.

Microscopic corrosion morphologies of B10 copper sheets after corroding for different times under oxygenated stirring and stationary conditions are shown in Figure 5 and Figure 6. Under stationary test conditions, a large number of corrosion spots appeared on the B10 surface during the marine full-immersion test, and with the progression of corrosion reaction, the corrosion product film on sample surface thickened continuously, with uniform and dense inner layer and loose corrosion product outer layer. There were many uneven corrosion pits on the sample surface. Under oxygenated stirring conditions, oxygen content was relatively high on the sample surface, and with the extension of corrosion time, small amounts of Mg, Cl, Ca and other elements gradually began to appear, which could reflect the gradual formation and increase in content of oxidation products on sample surface. Outer layer of corrosion products was loose and porous, which was easily detached from the sample sheet during sampling or solution replacement. In addition to the patchy corrosion morphology, signs of pitting corrosion were also visible on the samples.

Under both conditions, the corrosion product films on sample surfaces had cracking and local exfoliation. This may be because the substrates under corrosion product film were located at the grain boundaries, twins or other defects, and due to structural and compositional changes, the growth rate of corrosion product films in these places was slower than the growth rate of inner crystal films. It may also be the greater internal stress of corrosion product film than the film strength that caused cracking of films.

### 4.2. Effect of Medium Temperature

In order to study the effect of medium temperature on the corrosion behavior of B10 alloy, the control groups where medium temperatures were 45 °C and 60 °C were set up on the basis of the above tests, while all the other test conditions remained unchanged.

**Figure 7:**
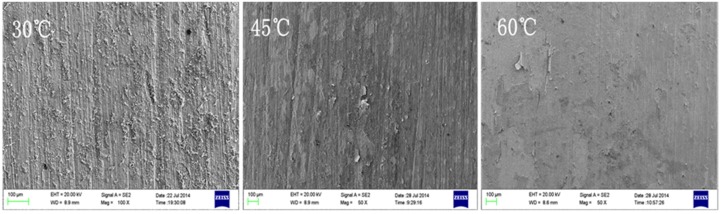
Microscopic corrosion morphologies of B10 alloy after one week of corrosion under different temperature conditions.

**Figure 8:**
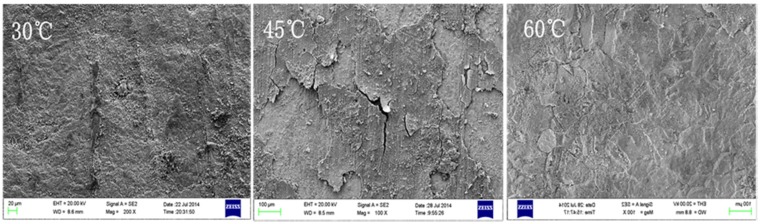
Microscopic corrosion morphologies of B10 alloy after four weeks of corrosion under different temperature conditions.

As can be seen from Figure 7 and Figure 8, on the first week, many particulate corrosion products were attached to the 30 °C sample surface, while at 45 °C and 60 °C conditions, a thin layer of corrosion product film had already been formed on the sample surfaces. With the increase of corrosion time, on the fourth week, uneven corroded substrate was visible in the sample at 30 °C conditions, with obvious pits; at 45 °C conditions, the surface corrosion product film portion of the sample began to rupture and exfoliate, and the revealed substrate was intact, without obvious corrosion pits; in comparison, at 60 °C conditions, the surface corrosion product film of sample had the smallest rupture, and the film and substrate were well bonded. 

## 5. Corrosion Prediction and Discussion

### 5.1. Corrosion Prediction Considering Dissolved Oxygen

**Figure 9:**
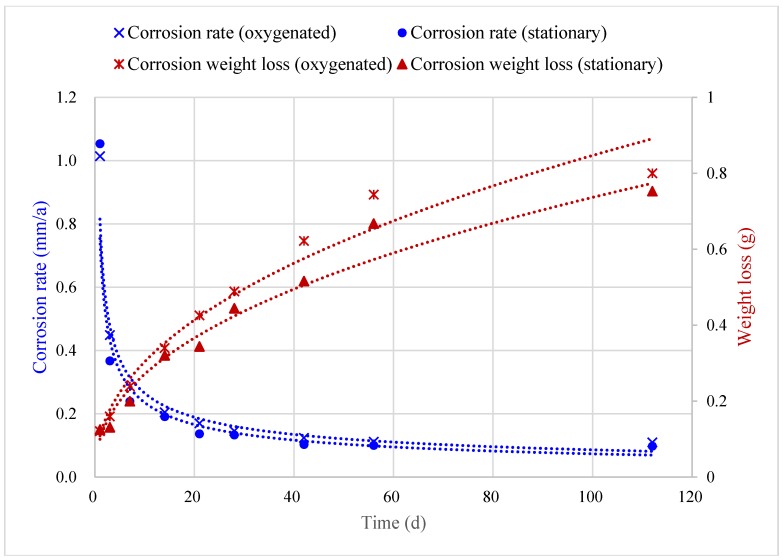
Changes in corrosion rate of B10 copper-nickel alloy over time.

Figure 9 shows the trends of changes in B10 copper corrosion rate under oxygenated stirring and stationary conditions over time. On the premise that the other test conditions were fixed, B10 alloy corrosion rate was markedly higher under the oxygenated stirring conditions than the stationary conditions. However, with the increase of corrosion time, the corrosion rates under both conditions gradually declined and leveled off.

Corrosion rates under full cycle test conditions were fitted using power function, which has been proven to be effective and accurate for corrosion study [15]. The corrosion rate formulas under oxygenated stirring and stationary conditions were:

(1) $$\begin{array}{l} v_{stirring}=1.013t^{-0.653},R^{2}=0.992\left(Oxygenated\right)\\ v_{stationary}=0.995t^{-0.653},R^{2}=0.984\left(Stationary\right) \end{array}$$

where $R^{2}$ was the goodness of fit coefficient.

From the perspective of corrosion weight loss, the corrosion weight loss models under oxygenated stirring and stationary conditions were:

(2) $$\begin{array}{l} w_{stirring}=0.121t^{0.419},R^{2}=0.981\left(Oxygenated\right)\\ w_{stationary}=0.108t^{0.419},R^{2}=0.983\left(Stationary\right) \end{array}$$

Let the corrosion weight losses, w, in the two equations equal, the following conversion relation could then be obtained:

(3) $$\begin{array}{l} 0.121\cdot t_{stirring}^{0.\text{419}}=0.108\cdot t_{stationary}^{0.419}\\ t_{stationary}/t_{stirring}=1.31 \end{array}$$

where $t_{stirring}$ is the number of corrosion days under oxygenated stirring conditions and $t_{stationary}$ is the number of corrosion days under stationary conditions.

Thus, one-day corrosion weight loss under oxygenated stirring conditions was equivalent to the 1.31-day weight loss under stationary conditions, while the corrosion rate under oxygenated conditions was 1.31 times higher than that under stationary conditions, so the corrosion rate acceleration factor was α = 1.31 under oxygenated conditions.

Based on the above results, the rate and quantity of corrosion under oxygenated and stationary conditions could be predicted separately. Corrosion quantity under oxygenated conditions could also be obtained according to the acceleration factor given known corrosion quantity under stationary conditions, and *vice versa*.

### 5.2. Corrosion Prediction Considering Medium Temperature

**Figure 10:**
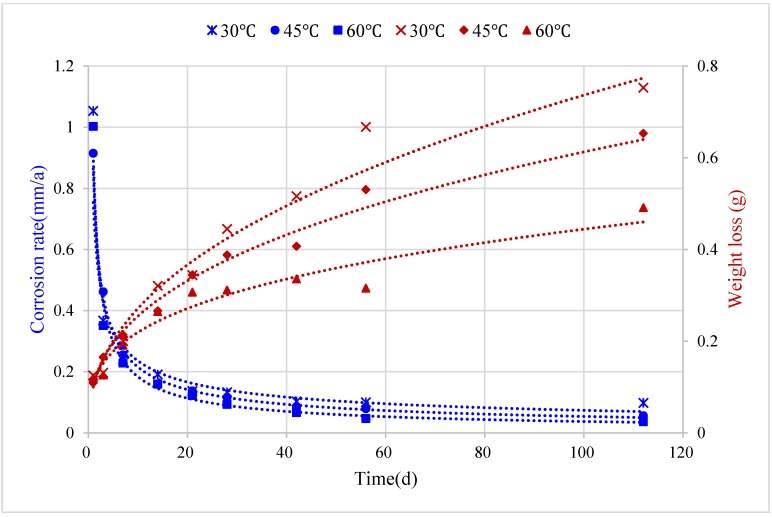
Corrosion rates of B10 copper sheet under different conditions.

Corrosion weight loss and corrosion rate of B10 copper sheet under different medium temperature conditions were determined by weight loss method, and their changes over time are shown in Figure 10. Average corrosion rate of B10 copper sheet at different medium temperatures exhibited a decreasing trend over the corrosion time. Corrosion rate during the initial two weeks of test was far higher than that after the two weeks. After four weeks of full immersion, the corrosion rate stabilized.

It can be seen from the trends of changes in the average corrosion rate of B10 copper sheet under different temperature conditions over corrosion time that at the beginning of the reaction, the higher the temperature, the greater the corrosion rate of B10 copper sheet. As shown in Figure 10, on the first day of corrosion, the corrosion rate was the highest for the sample under 60 °C conditions. Over time, after stabilization of the corrosion reaction, the average corrosion rate of B10 copper sheet decreased with rising temperature. After four weeks of corrosion, samples in the figure basically maintained a trend of fastest corrosion at 30 °C, followed by 45 °C, and slowest corrosion at 60 °C.

Corrosion rates of the entire cycle under different test conditions were fitted using power function [15], and the test results under highest and lowest temperatures conditions were selected for comparison. Corrosion rate formulas under various temperature conditions were as follows:

(4) $$\begin{array}{l} v_{30℃}=1.020t^{-0.708},R^{2}=0.986\\ v_{45℃}=0.955t^{-0.745},R^{2}=0.992\\ v_{60℃}=0.985t^{-0.774},R^{2}=0.995 \end{array}$$

From the perspective of corrosion weight loss, the corrosion weight loss models under corresponding conditions were as follows:

(5) $$\begin{array}{l} w_{30℃}=0.098t^{0.445},R^{2}=0.984\\ w_{45℃}=0.100t^{0.398},R^{2}=0.984\\ w_{60℃}=0.108t^{0.310},R^{2}=0.969 \end{array}$$

where *R*^2^ was the goodness of fit of the equations.

Corrosion rate and quantity at different temperatures could be predicted according to the above models to obtain the specific corrosion quantity and rate values at a certain time in the future, as well as the time required for reaching the maximum corrosion quantity.

There were a variety of factors attributable to the decrease in average corrosion rate of B10 copper sheet with increasing temperature. The phenomenon was mainly associated with the dissolved oxygen and the sample surface film. High temperatures could accelerate the decomposition of hydrogen peroxide, on the first day of corrosion, seawater under high temperature conditions contained rich dissolved oxygen, moreover, high temperatures would also accelerate gas diffusion, allowing the corrosion rate of B10 sample at 60 °C for one day far higher than that under other conditions. As the corrosion reaction proceeded, the dissolved oxygen content in high temperature seawater declined rapidly. Additionally, H_2_O_2_ contributed to the passivation of B10 copper sheet surface while catalyzing and accelerating the cathodic depolarization, thereby thickening the corrosion product film on sample surface, and hindering the corrosion reaction.

## 6. Conclusions

Summarily, it could be seen from the presentation above that Cu_2_O film thickens gradually with the extension of marine corrosion time. Its corrosion product is Cu_2_(OH)_3_Cl, which increases in quantity over time. Cl^−^ in it is the major corrosive factor responsible for the marine corrosion of copper and copper alloy.

Comparative analysis of tests between oxygenated stirring and stationary groups find that the corrosion morphology and products are very similar between two test conditions, except that the corrosion degree of B10 copper sheet is severer under oxygenated stirring conditions than under the stationary conditions. The acceleration factor of oxygenated stirring conditions with respect to the stationary conditions is 1.31. Oxygenated stirring during the accelerated degradation test can achieve better acceleration effect.

Based on the experimental results, corrosion prediction models under different environmental conditions were constructed. More specifically, corrosion medium temperature has a significant effect on the corrosion of B10 copper sheet. Corrosion rate is quantitatively measured after observing the stabilization of corrosion from the microscopic morphological perspective. With the increase in corrosion medium temperature, the corrosion rate decreases continuously, which is consistent with other copper-nickel alloy.
